# CHD1L in cancer and beyond: structure, oncogenic functions, and therapeutic potential

**DOI:** 10.1186/s13046-025-03428-1

**Published:** 2025-05-30

**Authors:** Sophia Clune, Paul Awolade, Hector Esquer, Qiong Zhou, Daniel V. LaBarbera

**Affiliations:** 1 Skaggs School of Pharmacy and Pharmaceutical Sciences, Department of Pharmaceutical Sciences, University of Colorado Anschutz Medical Campus, Aurora, CO 80045 USA; 2 Center for Drug Discovery, University of Colorado Anschutz Medical Campus, Aurora, CO 80045 USA; 3 University of Colorado Cancer Center, University of Colorado Anschutz Medical Campus, Aurora, CO 80045 USA

**Keywords:** CHD1L, ALC1, Chromatin remodeler, Oncogene, PARthanatos, DNA damage response, Cancer cell survival, Therapeutic target, CHD1L inhibitor (CHD1Li), Metastasis, Immune evasion

## Abstract

Chromodomain Helicase DNA-binding protein 1-Like (CHD1L) is a chromatin remodeling enzyme increasingly recognized as an oncogenic factor promoting tumor progression and metastatic potential by orchestrating transcriptional programs that drive epithelial-mesenchymal transition (EMT), cytoskeletal remodeling, and metastatic dissemination. In parallel, CHD1L has emerged as a master regulator of tumor cell survival by regulating DNA damage response and repair and enforcing G1 cell cycle progression. Furthermore, CHD1L plays a key role in immune evasion pathways by regulating signaling cascades and by suppressing both apoptotic and non-apoptotic cell death. In particular, CHD1L is a key suppressor of PARthanatos, a caspase-independent mechanism triggered by poly(ADP-ribose) (PAR) polymer fragmentation and apoptosis-inducing factor (AIF) activation. By regulating SPOCK1, MDM2, and TCTP, CHD1L further supports survival under cellular stress. Its overexpression correlates with metastasis, therapy resistance, and poor prognosis across many solid tumors. This review covers CHD1L’s structure, oncogenic functions, and developmental origins, and highlights emerging therapeutic strategies that target CHD1L as a druggable vulnerability in cancer.

## Background

The chromodomain helicase DNA-binding (CHD) family of genes plays an integral role in DNA homeostasis. Due to the complex packaging of chromatin, the nucleosomes must be reorganized by chromatin remodelers to allow other enzymes access to DNA, and members of the CHD family are major mediators of this process. Their ATP-dependent activity enables other nuclear proteins to bind DNA and perform critical genomic functions including DNA maintenance, transcription, repair, and synthesis [1].

Among the CHD proteins, CHD1L (chromodomain helicase DNA binding protein 1-like) confers the greatest selective growth advantage when overexpressed, as it increases proliferation and survival capacity significantly more than other members of the CHD family [2]. Originally named Amplified in Liver Cancer 1 (ALC1), *CHD1L* is located on the 1q21 chromosomal region, which is the most amplified region in hepatocellular carcinoma [3–5]. Since its discovery, CHD1L has been further identified as a key biomarker and oncogenic protein in many cancers, including colorectal, breast, bladder, lung, and kidney cancers, indicating its crucial role in cancer progression [2, 6–8].

Cancer is the second leading cause of death worldwide and imposes a substantial burden on healthcare systems across both developed and developing nations [9, 10]. The lifetime probability of any individual being diagnosed with malignant cancer is roughly 40%, and the 5-year survival rate for all cancer patients is currently 69%. This statistic represents a remarkable increase from a 49% survival rate 50 years ago, owing to advances in early screening and the development of targeted therapy [10]. Conventional cancer treatment includes surgical resection, chemotherapy, and radiation therapy, although these come with risks of significant complications and adverse effects [11]. However, targeted therapies aim to inhibit oncogenic proteins or signaling pathways that are aberrant in tumor cells, thereby limiting effects on normal tissues [12]. Despite advances, many emerging or approved targeted drug therapies continue to show limited overall response rates, and multidrug resistance (MDR) remains a persistent obstacle, underscoring the need for novel therapeutic strategies [13]. CHD1L has emerged as a promising molecular target to address this therapeutic gap due to its widespread overexpression and oncogenic activity across multiple types of cancer. Furthermore, it functions as a master regulator of cancer cell survival, promoting MDR to standard-of-care (SOC) therapies, including chemotherapy and other targeted agents.

The versatile function of CHD1L in cancer cell survival distinguishes this chromatin remodeler as both a prognostic biomarker and a prime molecular target for anticancer therapy. Experimental studies have shown that knockdown of CHD1L in cancer cell lines reduces tumor growth and increases sensitivity to both chemotherapy and targeted therapy [14–17]. These findings have prompted the development of CHD1L inhibitors as potential therapeutics to treat cancer [18–20].

This review provides a comprehensive analysis of CHD1L, organized around its structural features, molecular mechanisms, and roles in development and disease. As a key regulator of chromatin remodeling, DNA repair, and transcriptional control, CHD1L is essential for maintaining cellular homeostasis. Its dysregulation has been implicated in a wide range of conditions, including cancer, congenital disorders, neurological, cardiovascular, and viral diseases. However, this review places a primary focus on CHD1L’s role in cancer, where its overexpression and biochemical activity drive tumor progression, metastatic potential, therapeutic resistance, and poor clinical outcomes. We conclude by highlighting emerging therapeutic strategies targeting CHD1L as a novel and druggable oncogenic driver.

## Structural biology of CHD1L

### Homology to CHD1

The CHD1L protein is a chromatin remodeling enzyme whose structure reveals unique regulatory features distinguishing it from other CHD family members. The CHD family exists under the umbrella of the SNF-2 (sucrose non-fermentation 2) superfamily, a diverse group of ATP-dependent chromatin remodeling enzymes, which regulate and remodel tightly packed chromatin [5, 21]. There are two highly conserved signature motifs characteristic of the CHD family: N-terminal tandem chromodomains and a centrally located SNF2-like ATPase domain (SNF2-N) [22, 23]. The CHD N-terminal chromodomain directly binds methylated histones, while the ATPase domain hydrolyzes ATP and utilizes this energy to translocate histones along DNA [24–26]. In addition to their SNF2-N domain, CHD1 and CHD2 subtypes also contain a DNA binding domain located closer to the C-terminus, which is a helicase superfamily domain (HELICc).

**Fig. 1:**
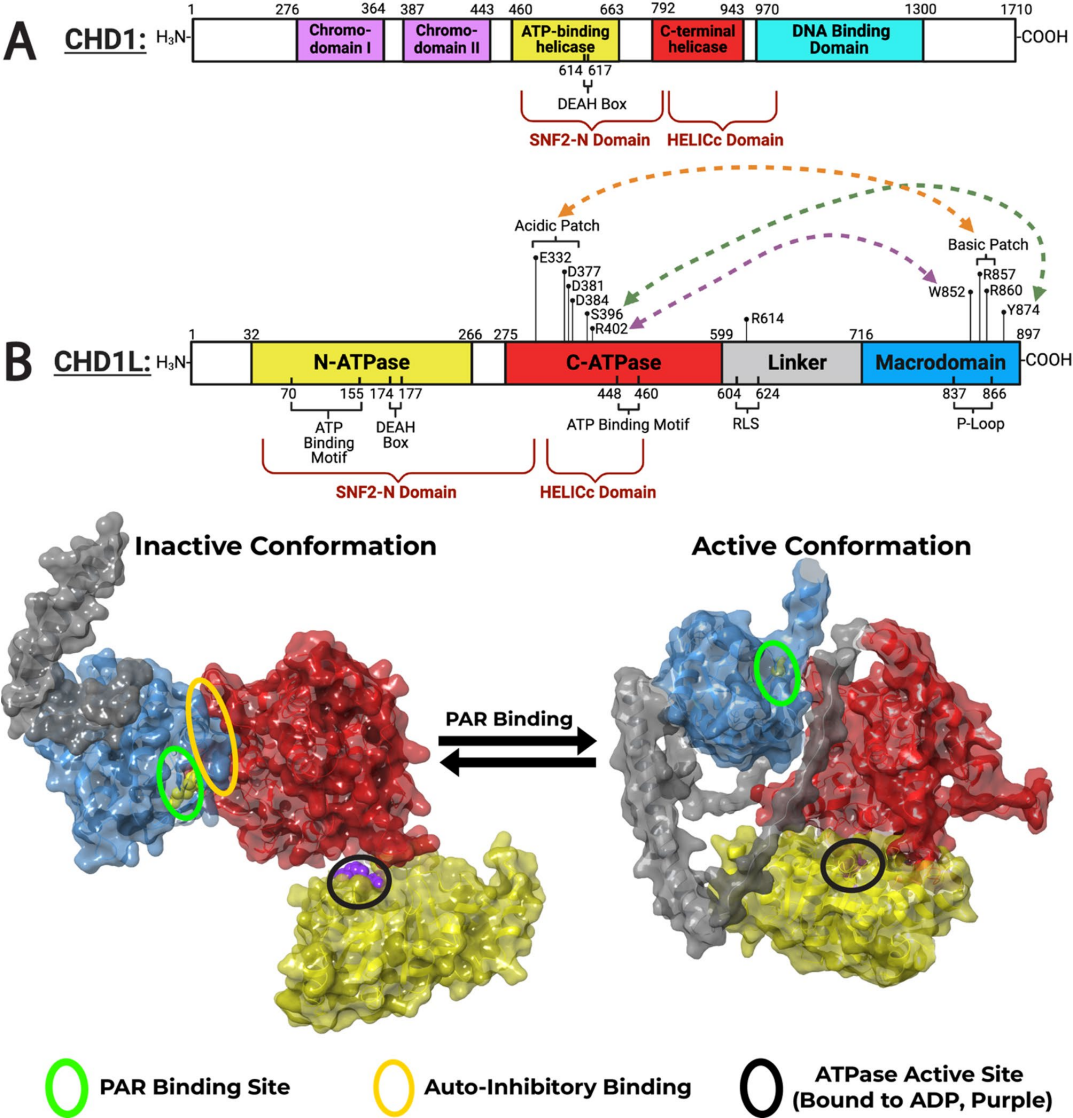
CHD1L structural homology to CHD1 and its autoinhibited state. (**A**) Linear domain architecture of CHD1, highlighting structural domains (colored boxes), sequence-defined motifs (red text), and key amino acid residues. (**B**) Domain schematic of CHD1L (top) and surface models of the autoinhibited (left) and active (right) conformations (bottom). Structural domains are color-coded: N-ATPase (yellow), C-ATPase (red), linker (gray), and macrodomain (blue). Predicted intramolecular interactions are shown as colored dashed arrows. In the autoinhibited state, the macrodomain binds the C-ATPase lobe (yellow oval), blocking the ATPase active site (black circle) and PAR binding site (white circle). PAR binding relieves this interaction, allowing ATPase domain realignment and activation. Structures were generated using PDB: 7EPU (inactive) and AlphaFold (active) in Schrödinger Maestro

There are 9 subtypes of the CHD family, which are numbered CHD1–9, and CHD1L is named as such because of its high similarity to CHD1, particularly within the helicase domain [22, 27]. The sequence homology between the SNF2-N domain of CHD1 and CHD1L is 45%, while the HELICc domains are 59% homologous (Fig. 1A) [5]. However, CHD1L is distinct from its namesake counterpart due to the presence of a C-terminal macrodomain and a lack of tandem chromodomains, as shown in the comparative domain schematic in Fig. 1B. Given that CHD proteins are specified by the presence of tandem repeats, CHD1L is not technically a part of the CHD family and instead is classified as a member of the SNF2-like superfamily [28].

Although homologous, the respective domains of CHD1 and CHD1L do not function identically. The SNF2-N domain of CHD1 contains the helicase-like ATPase motor, which provides the force for nucleosome movement along the DNA strand, while the DNA binding domain facilitates enzyme–DNA–nucleosome interactions necessary for chromatin remodeling [29]. CHD1’s double chromodomain works by recognizing sites of lysine methylation on histone tails, and also functions as an autoinhibitory domain, as it binds to and regulates the activation of the ATPase motor [24, 30]. Meanwhile, CHD1L’s sequentially identified SNF2-N domain and helicase superfamily C-terminal domain (HELICc) approximately correspond to the structurally identified N- and C-ATPase lobes, respectively, which collectively constitute the catalytic region of the protein (Fig. 1B) [5, 31, 32]. In lieu of a regulatory tandem chromodomain, CHD1L’s activity is regulated by the autoinhibitory macrodomain.

### CHD1L domain architecture and autoinhibition

The CHD1L gene is located at the 1q21.1 chromosomal region and is 53,152 base pairs long [5]. The mRNA transcribed from this gene contains 2,890 base pairs and codes for an 897 amino acid product with a mass of 98 kDa. CHD1L is composed of four structurally identified domains: starting at the N-terminus, there are two ATPase domains designated the N-ATPase (residues 32–266) and C-ATPase (residues 275–599), a C-terminal macrodomain (residues 717–897), and a linker region (residues 600–716) that connects the macrodomain to the C-ATPase lobe (Fig. 1B) [33, 34]. In addition to domain annotations, the CHD1L protein crystal and AlphaFold structures provide insight into the spatial orientation of its functional domains, including the PAR binding sites, auto-inhibited binding, and the ATPase active site (Fig. 1B).

### Structural transitions between autoinhibited and active states

**Fig. 2:**
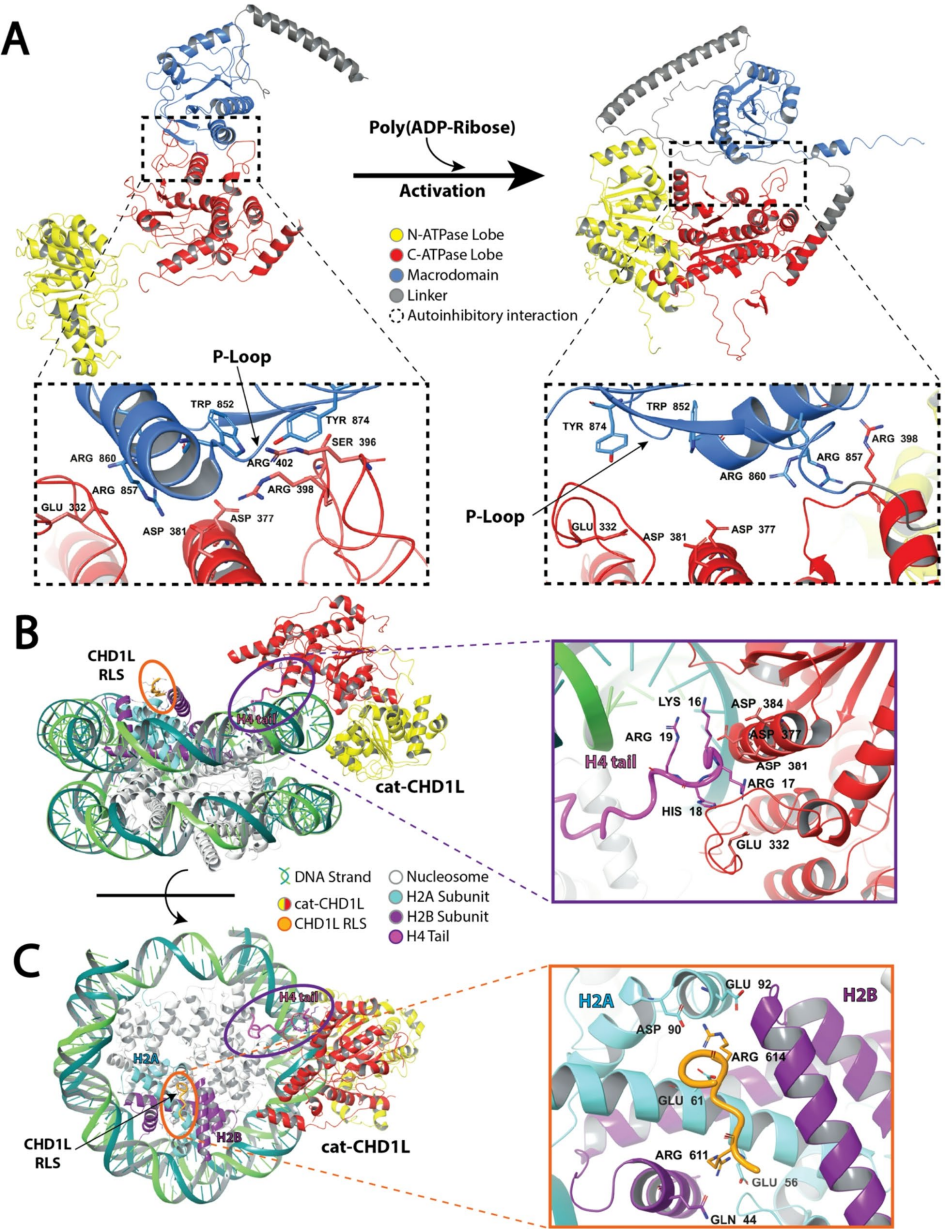
CHD1L conformational activation and nucleosome engagement. (**A**) Ribbon representations of full-length CHD1L in the autoinhibited state (left) and activated state (right), showing the release of the macrodomain’s basic patch from the acidic patch on the C-ATPase lobe following PAR binding. This conformational change brings the N- and C-terminal ATPase lobes into closer proximity, enabling catalytic activity. Dashed boxes highlight close-up views of the autoinhibitory interface in each state, with key interacting residues labeled. (**B**) Side view of nucleosome-bound cat-CHD1L, showing a zoomed in view of its interaction with DNA, the H4 tail, and the linker region. The H4 tail and linker are shown as tubes, and colored circles indicate their positions. (**C**) Top view of cat-CHD1L bound to the nucleosome, with zoomed-in view of the interaction between the CHD1L regulatory linker sequence and the acidic patch formed by the H2A/H2B dimer. Structures shown in panels A–C were generated from PDB: 7EPU (panel A, left), AlphaFold (panel A, right), and PDB: 7ENN (panels B–C) using Schrödinger software

The two ATPase domains function together as an ATP-binding helicase motor and can adopt an open or closed conformation, corresponding to an inactive (Fig. 2A; **left**) or active state (Fig. 2A; **right**), respectively [31]. In the autoinhibited state, the macrodomain is pressed against the C-ATPase lobe, holding both ATPase domains in an open state and preventing catalytic activity. Arginine residues 860 and 857 within the basic patch of the macrodomain are responsible for this, as they participate in electrostatic interactions with positively charged residues at the acidic patch on the C-ATPase lobe (Figs. 1B and 2A). Following cellular cues leading to PAR binding, the macrodomain releases the ATPase domains from an autoinhibitory state, allowing CHD1L to carry out chromatin remodeling [32].

For example, after DNA damage occurs, poly(ADP-ribose) polymerase 1 and 2 (PARP1/2) synthesize poly(ADP-ribose) (PAR). PARP1/2-mediated synthesis of PAR chains lead to PARylation of itself and other proteins on chromatin and involved in the DNA damage response (DDR) [35]. PAR chains on activated PARP1/2 recruit CHD1L to sites of DNA damage, where they bind the CHD1L macrodomain at the P-loop and adjacent motifs (Fig. 2A). Specifically, during autoinhibition, the macrodomain residues Trp852 and Tyr874 interact with Arg398/402 and Ser396 residues of the C-ATPase lobe, respectively (Fig. 2A; **left**). However, these interactions are interrupted by PAR binding, inducing a conformational change in the protein’s structure, most significantly in residues 328–355 in the C-ATPase lobe and 604–639 in the linker region [31]. This change is indicative of the macrodomain ceasing contact with the C-ATPase domain to adopt a catalytically active state (Fig. 2A; **right**), where the ATPase lobes are in closer proximity. This conformational state facilitates chromatin remodeling fueled by ATP hydrolysis at the active site located in the N-ATPase lobe, which is catalytically competent only when aligned with the C-ATPase lobe [33].

The C-ATPase acidic residues that are released by the macrodomain—Glu332, Asp377, and Asp381—are critical for CHD1L activity, as they bind the H4 tail on nucleosomes (Fig. 2B) [33]. The H4 tail has a basic patch from residues 16–19, which engages with the acidic pocket on the C-ATPase. Notably, Asp381 participates in essential interactions for both autoinhibition and activation, as it forms electrostatic bonds with arginine in the macrodomain during dormancy and also binds the H4 tail on the nucleosome to initiate chromatin remodeling after activation (Fig. 2B; **zoomed region**).

Interestingly, this is not the only interaction required for activation. The linker region of CHD1L also binds the acidic patch on the nucleosome via an arginine anchor, Arg611 [34]. A subsection of the linker region known as the regulatory linker segment (RLS) consists of residues 604–624 and is a critical motif for CHD1L nucleosome binding (Fig. 2C). Arg611 acts as a tether to the acidic patch located on the entry side of the H2A/H2B dimer. This arginine anchor on the RLS projects into a pocket between alpha helices 1 and 2 of H2A (Fig. 2C; **zoomed region**) [34]. The first crystal structure of CHD1L indicated that Arg611 specifically interacts with Glu56 of H2A and Gln44 of H2B [33]. Furthermore, this study revealed that Arg614 is also a critical piece of the RLS, as it participates in hydrogen bonds with Glu61, Asp90, and Glu92 of H2A. The binding of the RLS to this acidic pocket within the histone stabilizes the active conformation of CHD1L and prevents it from transitioning back to its autoinhibited state [33, 36].

### Structural insights from CHD1L mutations and isoforms

Multiple lines of evidence, including site-directed mutagenesis and transcript variant analysis, have revealed key features of CHD1L’s regulatory mechanisms and chromatin remodeling function. Mutational studies have confirmed critical residues involved in autoinhibition and activation. For instance, mutations in macrodomain residues Arg860 and Arg857 disrupt electrostatic interactions with the C-ATPase lobe, resulting in constitutive CHD1L activity due to loss of autoinhibitory control (Fig. 2A) [31]. These residues are also found mutated in tumor samples, supporting their relevance to disease pathogenesis [37]. Similarly, mutations in Trp852 lead to hyperactivation of CHD1L independent of PAR binding or PARP1/2 activity, underscoring its role in macrodomain-mediated inhibition [33].

Residues within the linker region, specifically Arg611 and Arg614, are also critical for CHD1L function. Although these mutations do not affect ATPase activity, they impair chromatin remodeling by disrupting interactions between the RLS and the nucleosome acidic patch (Fig. 2C) [33, 34]. These contacts are essential for maintaining the active state of the enzyme. Additional mutational studies have revealed residues that modulate chromatin remodeling without altering ATPase activity. Arg260 and Arg319 mutants show a significant suppression of nucleosome sliding associated with a modest reduction in ATP hydrolysis [33]. Arg260 facilitates the interaction between the N- and C-ATPase lobes, while Arg319 faces the DNA strand and may participate in DNA engagement. Mutations at Arg260 also reduce CHD1L’s thermal stability. In contrast, mutation of Arg457 abolishes ATPase activity entirely, consistent with its proposed role as an “arginine finger” in catalysis.

In addition to point mutations, CHD1L function may be influenced by transcript diversity. At least six splice variants have been identified, each altering the inclusion of critical functional domains (Fig. 1B) [27]. The canonical isoform encodes a 897-residue protein with a complete ATPase motor and macrodomain. Variant 2 lacks residues 331–424, removing part of the C-ATPase lobe. Variant 3 deletes the first 113 residues, affecting the N-ATPase domain. Variants 4 and 5 have larger deletions within the N-terminal region, missing residues 44–246 and 43–246, respectively. Variant 6 is a non-coding transcript. Although their biological roles remain unknown, these isoforms may modulate DNA binding, ATPase activity, or regulatory interactions by altering domain structure and positioning. Together, structural mutations and isoform diversity highlight the finely tuned architecture of CHD1L and its importance in regulating chromatin remodeling and genome integrity.

## CHD1L in development and cancer

### Embryonic and neural development

**Fig. 3:**
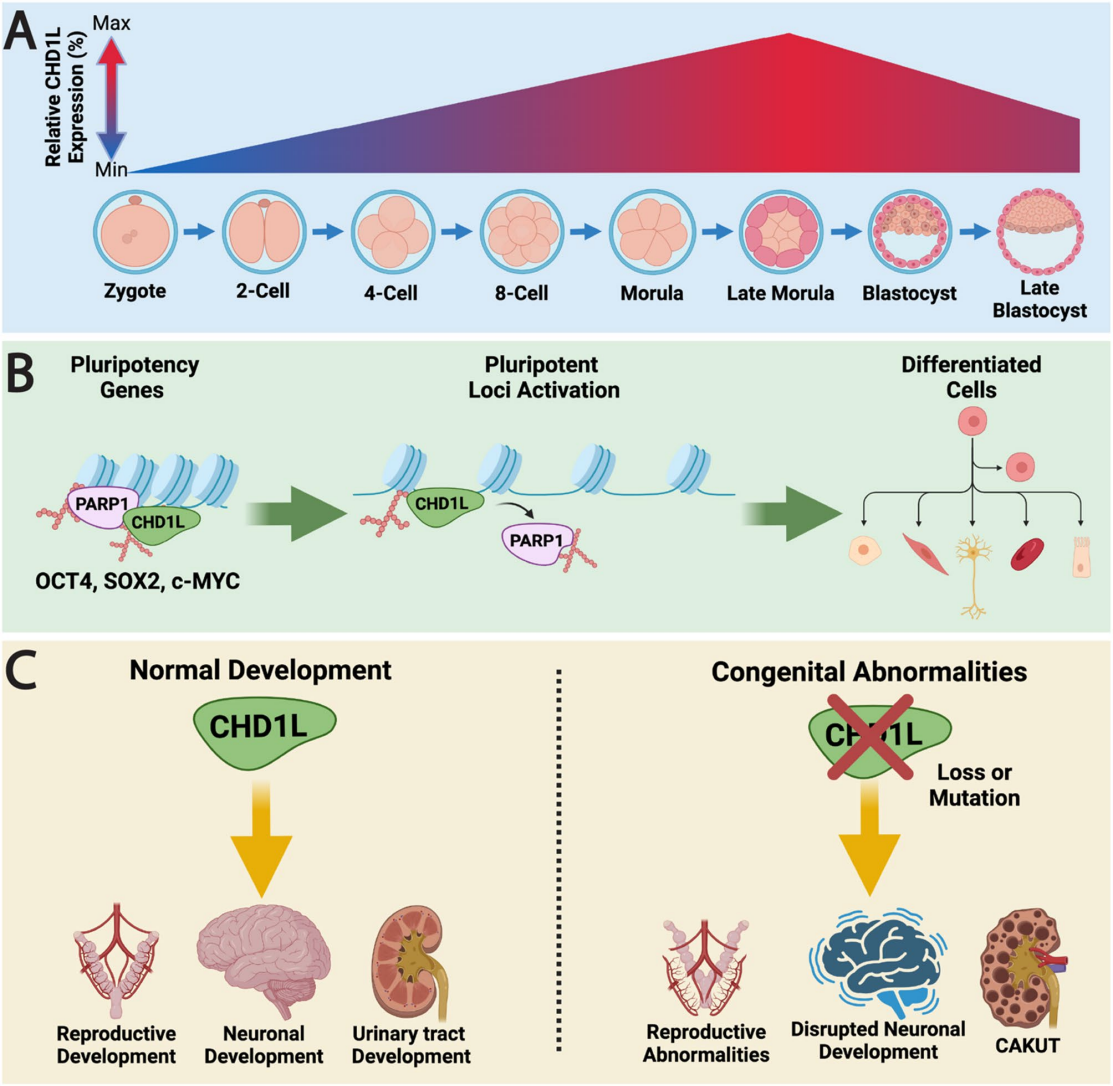
CHD1L function in early development and pluripotency. (**A**) CHD1L expression progressively increases during pre-implantation development, peaking at the late morula stage and declining through the blastocyst stage. (**B**) In stem cell progenitors, CHD1L is recruited by PARylated PARP1 to pluripotency loci, where it remodels chromatin to enhance DNA accessibility and activate key pluripotency genes, including OCT4, SOX2, and c-MYC. This activity promotes the establishment of a stem-like state critical for early developmental reprogramming. (**C**) CHD1L is highly expressed during organogenesis in the developing brain, kidney, and reproductive tract. Loss-of-function mutations or copy number variants affecting CHD1L result in embryonic lethality or cause congenital abnormalities, including CAKUT (congenital anomalies of the kidney and urinary tract), Müllerian duct anomalies (MDA), and neurodevelopmental delay

CHD1L plays a critical role in early embryonic development, where it orchestrates chromatin remodeling programs essential for cellular plasticity, pluripotency, and tissue morphogenesis. These developmental functions rely on CHD1L’s chromatin remodeling, a mechanism later exploited in cancer to drive transcriptional reprogramming and tumor progression. Prior to implantation, the zygote requires temporary totipotency to accomplish the extensive development ahead, and complex chromatin organization does not favor this state [38]. Thus, chromatin remodelers that increase DNA accessibility are upregulated in the pre-implantation embryo [39]. CHD1L expression peaks late in the morula stage of development and knockdown of CHD1L prior to the blastocyst formation will lead to developmental arrest (Fig. 3A). However, CHD1L expression was not necessary for the survival and proliferation of cultured embryonic stem cells.

The post translational modification of PARylation via PARP1 is also a critical regulator of cell reprogramming during development [40]. PARP1 induces stemness in embryonic stem cells by mediating activation of pluripotency genes such as OCT4, SOX2, KLF4, and c-MYC [41–43]. During this process, PARP1 recruits CHD1L to these pluripotent loci, suggesting its nucleosome remodeling function is key for the activation of these genes and cellular reprogramming towards a state of stemness (Fig. 3B) [44].

During embryogenesis, CHD1L is expressed in many growing organs, but mainly in the developing brain [45]. CHD1L has been associated with the expression of genes involved in the development of the ectoderm, the outermost layer that ultimately gives rise to the epidermis and nervous system [46–48]. CHD1L upregulates PAX6, in particular, a transcription factor critical to forebrain development [46, 49]. As such, when CHD1L knockdown occurs in human embryonic stem cells, neuroepithelial differentiation is compromised, validating its role in neural organogenesis. CHD1L is also highly expressed in the developing renal tract. Immunohistochemistry analysis of embryonic kidney tissue showed that CHD1L is significantly upregulated in the early uretic bud and nephron precursors during weeks 7–11 of embryonic development [45]. These premature structures will eventually become the collecting system, nephrons, glomerulus, and distal tubule. Based on these studies, CHD1L plays an important role in gestational organogenesis of the kidney and urinary tract, as well as the brain (Fig. 3C).

### Expression in the male reproductive system

Adult CHD1L expression is diminished in almost all tissues compared to corresponding fetal samples. However, the testes display the highest level of CHD1L expression out of all adult organs [28, 45]. The first association of CHD1L with the male reproduction system was made in 2010, when CHD1L was discovered to be abundantly expressed during sperm development in the accessory sex glands of *Eirocheir sinensis*, the Chinese mitten crab [50]. The upregulation of this chromatin remodeler was then observed in human testis through mRNA analysis, before the trend was confirmed in mouse models [45, 51]. Recently, CHD1L has also been identified as a key regulator of sperm production cycles in geese [52].

Immunostaining studies demonstrate that CHD1L is predominantly expressed in undifferentiated spermatogonia, stem cells that self-renew and mature into gametes through a process of spermatogenesis [51]. Upregulation of CHD1L is concurrent with the expression of OCT4, SOX2, and NANOG, other pluripotency regulators in spermatogonial stem cells (SSC) (Fig. 3B). These factors are targets of the glial cell line-derived neurotrophic factor (GDNF) signaling, which is critical to the survival and self-renewal of undifferentiated spermatogonia. Moreover, these genes are downregulated during CHD1L knockdown, suggesting that it acts as an important element within the GDNF signaling pathway within germ line stem cells [51].

It was later discovered that CHD1L regulates the stemness of spermatogonial cells through the microRNA (miRNA), miR-486, which is transcriptionally downregulated by CHD1L in SSCs [53]. miR-486 targets matrix metalloproteinase 2 (MMP2) mRNA for degradation, downregulating its expression in developing sperm cells. Because MMP2 activates β-catenin signaling, this knockdown in turn decreases expression of SSC stemness genes such as the GDNF targets, while increasing apoptosis and cell cycle arrest. This pathway appears to modulate spermatogenesis, as miR-486 decreases stemness and promotes differentiation of the SSCs.

### Congenital abnormalities

CHD1L plays a critical role in embryonic development and is essential for survival throughout gestation (Fig. 3A). Complete knockout of CHD1L results in early embryonic lethality, underscoring its fundamental role in early development. In contrast, missense mutations or structural variants in CHD1L have been identified in patients with a range of congenital abnormalities, particularly those affecting the renal and reproductive systems (Fig. 3C) [39, 44, 45].

CHD1L mutations have been recognized as a source of *c*ongenital *a*nomalies of the *k*idney and *u*rinary *t*ract (CAKUT). Several different heterozygous missense mutations have been identified in CAKUT patients, resulting in variable phenotypes including renal dysplasia, kidney malrotation, ureterovesical junction obstruction, and posterior urethral valves [54, 55]. Many of the CHD1L mutations associated with kidney defects were in or near the macrodomain and lead to decreased affinity for PARylated PARP1, a critical regulator of stemness during fetal development (Fig. 3B) [45]. CHD1L variants have also been observed as causative to congenital defects in the female reproductive tract, specifically Müllerian duct abnormalities (MDA) [56]. Although quite rare, CHD1L mutations are estimated to cause ~ 4% of MDA cases, which may lead to infertility and spontaneous miscarriages. Although not well understood, CHD1L’s role in cell migration has been proposed to mediate Müllerian duct formation, and a loss-of-function mutation may contribute to congenital defects (Fig. 3C.).

Copy number variants in the 1q21.1 locus, which contains the *CHD1L* gene, present even further reaching consequences. Numerous low copy repeats located on this locus may render this region vulnerable to microdeletions or microduplications via nonallelic homologous recombination [57]. The deletion of the 1q21.1 locus can lead to stillbirths wherein fetuses present with skeletal malformations [58]. However, 1q21.1 deletions have been reported as viable in some cases, although they are associated with developmental delay, microcephaly, facial abnormalities, seizures, oligohydramnios, and congenital heart anomalies [55]. Furthermore, duplications of this region cause overlapping symptoms such as developmental delays, dysmorphic facial features, and cardiac malformation, but may also lead to macrocephaly [59]. Additionally, *CHD1L* has been explored as a candidate gene for behavioral disorders such as ADHD (Attention Deficit/Hyperactivity Disorder) and autism, with microdeletions and microduplications at this locus linked to these conditions in children [60]. However, this chromosomal region also harbors *PRKAB2*, a key component of the AMP kinase complex, raising the possibility that these neurodevelopmental traits may result from alterations in *PRKAB2*, *CHD1L*, or their combined effects [61]. Consequently, the specific contribution of *CHD1L* to these disorders remains uncertain.

### CHD1L in cancer: expression and oncogenic function

Among all biological contexts in which CHD1L has been studied, its role in cancer is by far the most extensively characterized. CHD1L contributes to many hallmarks of cancer, including enhanced survival, evasion of programmed cell death, metastatic potential, evasion of the immune response, and MDR. CHD1L is overexpressed in a wide range of malignancies, and this overexpression is strongly associated with tumor progression, metastatic disease, and poor clinical outcomes for breast, ovarian, bladder, lung, colorectal, and many other cancer indications [2, 4, 6, 8, 18, 62–67].

CHD1L has a variety of oncogenic functions that promote tumor progression, metastatic potential, cancer cell survival, and resistance to treatment [28]. Clinical studies show that CHD1L is associated with more aggressive cancers and poor prognosis across multiple cancer types. This correlation has been extensively studied in gastrointestinal tumors but is also observed in other cancers [67–69]. Studies of patient tumor samples report CHD1L overexpression in breast, pancreatic, non-small cell lung, and many other cancer indications [62, 64, 65, 67, 70] (Table 1).

**Table 1:**
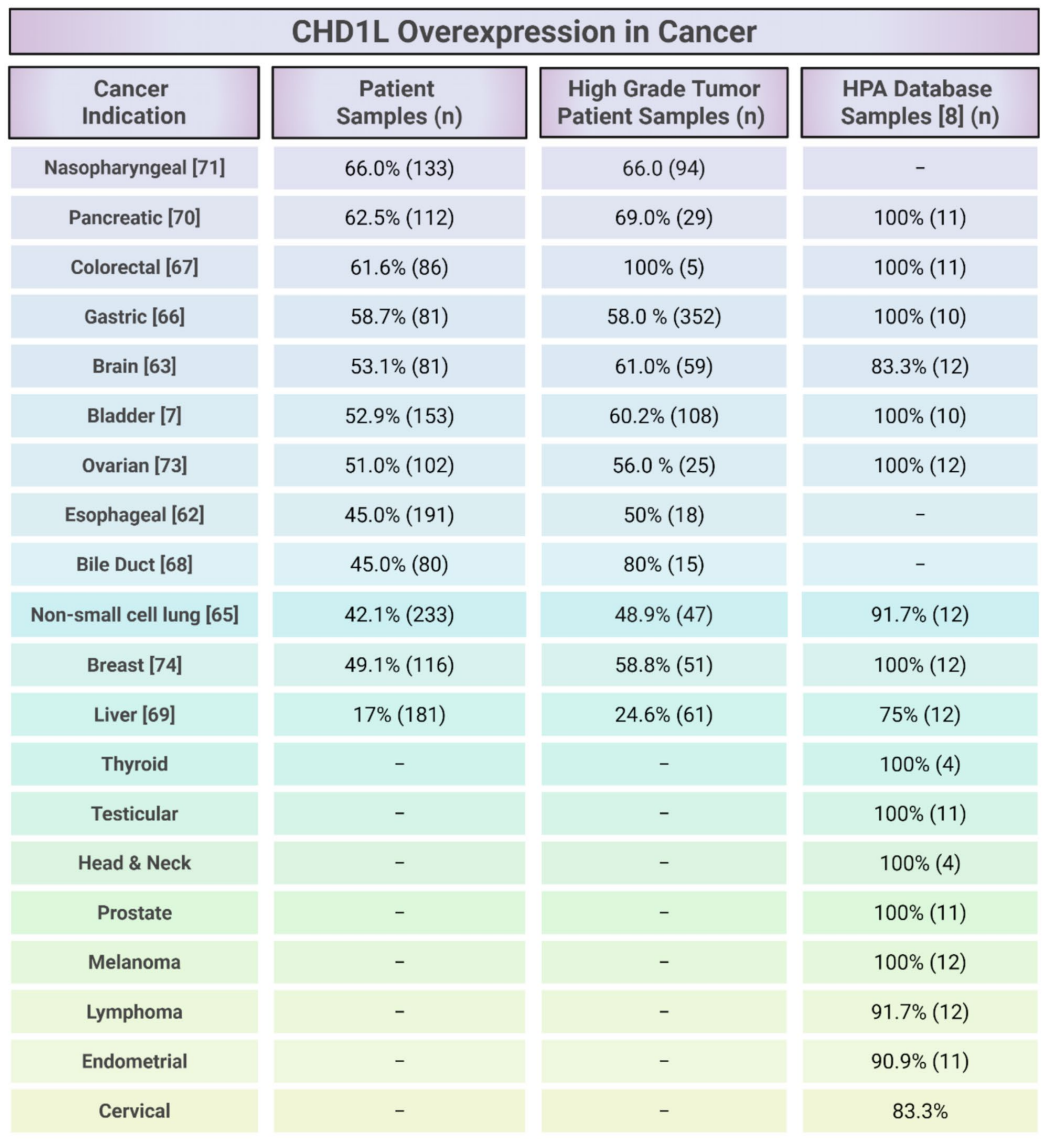
CHD1L overexpression levels across different cancer indications CHD1L expression was determined by immunohistochemistry analysis from independent studies of patient sample pools or as described from the Human Protein Atlas (HPA) [7, 8, 62, 63, 65–73]

A recent pan-cancer analysis comparing CHD1L expression in normal and tumor tissues suggests its overexpression may approach 100% in several cancers, including thyroid, colorectal, stomach, head and neck, prostate, ovarian, and skin cancers [8] (Table 1). Despite some variability in prevalence across studies, all findings consistently link CHD1L overexpression with worse clinical outcomes. Higher levels of CHD1L are associated with increased tumor volume and grade, as well as lower overall median survival time [6, 18, 70, 72]. However, CHD1L’s oncogenic role is not solely driven by its expression level; many cancers, regardless of CHD1L expression level, appear to rely on its biochemical activity to sustain cancer cell survival and progression [18, 74–76].

CHD1L is also strongly associated with metastatic disease. Expression levels are significantly increased in metastatic lesions compared to primary tumors, and CHD1L has been identified as an independent prognostic biomarker of metastatic potential [6, 66, 72]. Moreover, knockdown of CHD1L dramatically reduces the ability of tumor cells to proliferate, migrate, and invade neighboring tissues, all critical steps in the metastatic process [64, 70, 77]. These findings demonstrate that CHD1L plays a significant functional role in regulating epithelial–mesenchymal transition (EMT) and metastatic potential in cancer [4, 18, 20, 64, 65, 68, 78, 79].

Another significant contributing factor to CHD1L’s oncogenic potential is its role in therapy resistance. High CHD1L levels promote MDR to chemotherapy and a variety of other targeted therapies [2, 15–18, 74–76]. Proposed mechanisms include the upregulation of drug efflux pumps, inhibition of apoptosis, enhancement of DNA repair capacity, modulation of metabolic pathways, and reinforcement of cell motility signaling pathways such as EMT [17, 18, 80, 81]. CHD1L has also been shown to suppress a non-apoptotic form of programmed cell death known as PARthanatos [75, 76]. This suppression contributes to therapy resistance by enabling tumor cells to evade both apoptotic and non-apoptotic cell death. Notably, knockdown or inhibition of CHD1L consistently sensitizes tumor cells to chemotherapy and other targeted therapies, even in previously resistant models, positioning CHD1L as a compelling target to overcome MDR in a multitude of cancers [2, 16–18, 75, 76]. This comprehensive body of evidence has sparked increasing interest in the development of therapeutic strategies to target CHD1L in cancer.

## Molecular functions of oncogenic CHD1L

**Fig. 4:**
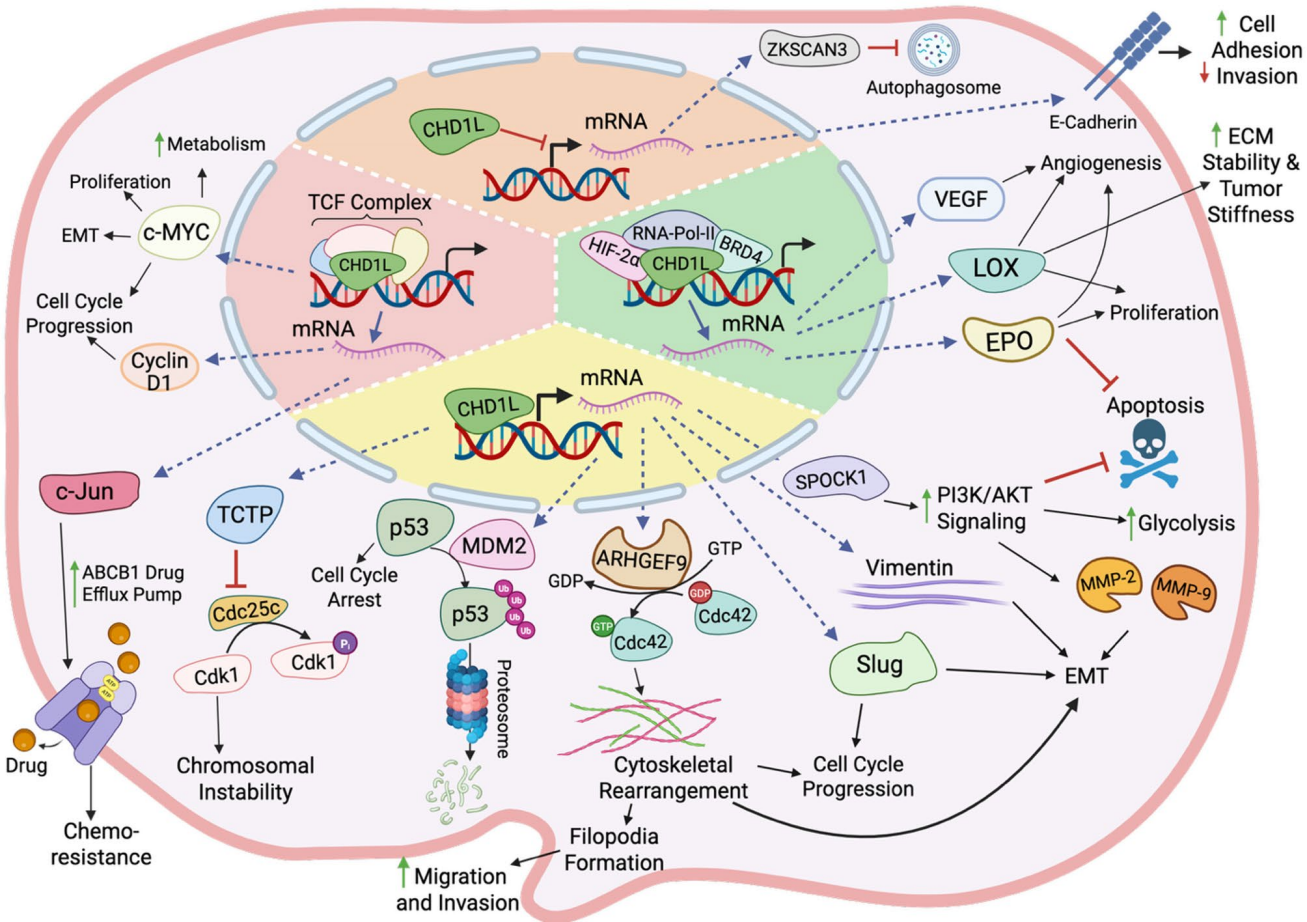
CHD1L Regulation of transcription, oncogenic signaling, and programmed cell death. CHD1L regulates gene expression through ATP-dependent chromatin remodeling at key genomic loci, driving oncogenic programs across multiple signaling axes. In the nucleus, CHD1L activates transcription of oncogenes that promote proliferation (Cyclin D1), survival (TCTP, MDM2), multidrug resistance (ABCB1), motility (ARHGEF9, Cdc42), and immune evasion (c-MYC, c-JUN) by cooperating with transcriptional complexes such as TCF/LEF (**red quadrant**) and hypoxia-inducible pathways involving HIF-2α (**green quadrant**). These programs converge on EMT, angiogenesis, therapy resistance, proliferation, and cancer cell survival. CHD1L also represses tumor suppressor genes, including E-cadherin (**orange quadrant**), and interacts with ZKSCAN3 to modulate autophagy. It regulates cytoskeletal rearrangement, metastatic potential, and apoptosis suppression via the SPOCK1–PI3K–AKT signaling axis, as well as invasion through ARHGEF9–Cdc42 signaling (**yellow quadrant**). CHD1L-mediated transcription of MDM2 leads to ubiquitin-mediated degradation and inhibition of p53, suppressing apoptosis and cell cycle arrest. Collectively, these activities promote immune evasion, therapy resistance, proliferation, cancer cell survival, and increased metastatic potential

CHD1L regulates cellular homeostasis through dynamic control of nucleosome architecture [82, 83]. Its recruitment to chromatin is often regulated by PAR-mediated signaling in the nucleus, linking its activity to genome surveillance and repair [84, 85]. Beyond this canonical role, CHD1L contributes to the transcriptional regulation of genes involved in key cellular programs that guide embryonic development and tissue organization, which are programs frequently hijacked in human disease (Fig. 4) [53, 76, 78, 86, 87]. CHD1L is not a classical transcription factor; rather, it promotes transcription through ATP-dependent chromatin remodeling at promoter and enhancer regions. By integrating environmental cues and intracellular stress signals, CHD1L modulates chromatin accessibility to regulate pathways essential for cellular plasticity, lineage commitment, and survival under stress [8].

### Control of gene expression and signaling

One of the most well-characterized roles of CHD1L-mediated transcriptional regulation is its promotion of epithelial-mesenchymal transition (EMT), a process by which epithelial cells lose polarity and adhesion, adopting mesenchymal traits (Fig. 4) [88, 89]. This transition is associated with increased cellular plasticity, altered differentiation states, and enhanced resistance to environmental and intracellular stress. EMT is often characterized by altered expression of phenotypic biomarkers. Common epithelial markers include E-cadherin, while mesenchymal markers include vimentin, N-cadherin, and slug (SNAI2) [90, 91]. In epithelial cells, E-cadherin mediates cell–cell adhesion, whereas vimentin, an intermediate filament protein, and slug, a transcription factor, are hallmarks of the mesenchymal phenotype [91]. Furthermore, aberrant EMT favoring quasi-mesenchymal cell phenotypes is associated with both malignant disease in cancer and in organ fibrosis [90, 92]. Upregulation of CHD1L in cancer cells correlates with increased vimentin and slug expression, along with decreased E-cadherin, suggesting a shift toward EMT. Conversely, CHD1L knockdown upregulates E-cadherin and suppresses vimentin and N-cadherin, indicating EMT reversal and loss of cancer stem cell traits [18, 63]. Numerous literature reports demonstrate that CHD1L drives EMT-associated transcriptional programs that promote epithelial plasticity and contribute to disease progression [4, 18, 20, 27, 28, 53, 63–65, 68, 72–74, 76, 77, 93, 94].

In addition to its role in EMT, CHD1L regulates cytoskeletal remodeling, metabolic reprogramming, and adaptation to microenvironmental stress, promoting cell motility and invasion (Fig. 4). For example, CHD1L upregulates expression of ARHGEF9, a guanine nucleotide exchange factor for the Rho GTPase Cdc42, which drives actin filament assembly and promotes cell motility [4]. CHD1L also directly regulates and increases SPOCK1 expression, a matricellular glycoprotein that activates the PI3K/Akt/mTOR signaling pathway, suppressing apoptosis and promoting metabolic activity via downstream phosphorylation of Akt, ARK5, and mTOR [64, 78, 81, 95]. This pathway induces matrix metalloproteinases (MMP-2 and MMP-9), which degrade basement membranes and facilitate migration and invasion [96]. In mouse models, CHD1L knockdown or PI3K pathway inhibition reduced metastasis, highlighting the role of this axis in tumor progression [64]. CHD1L directly promotes the expression of TCTP (translationally controlled tumor protein), contributing to tumor cell growth and enhanced survival [97]. CHD1L also regulates HIF-2α transcription and downstream hypoxia-responsive genes, including VEGFA, LOX, EPO, and NDNF (Fig. 4) [2]. Through regulation of ARHGEF9, SPOCK1, and hypoxia-responsive targets, CHD1L orchestrates a transcriptional network that promotes cell motility, invasion, adaptation to cellular stress, and survival.

One of the best-characterized pathways influenced by CHD1L is the Wnt-responsive TCF/LEF transcriptional complex, which regulates gene expression programs involved in EMT, stemness, and survival (Fig. 4) [98, 99]. CHD1L physically associates with the TCF/LEF complex and is enriched at Wnt-responsive elements [18]. Its recruitment facilitates chromatin accessibility and transcriptional activation of canonical TCF/LEF target genes such as c-MYC, Cyclin D1, and c-Jun [18, 99–101]. CHD1L also regulates ABCB1 expression, an efflux pump that exports metabolic byproducts, environmental toxins, lipophilic compounds, and drug therapies, by enhancing transcription of c-Jun, a TCF/LEF target gene. Increased c-Jun levels upregulate ABCB1, linking CHD1L activity to gene programs involved in efflux and stress adaptation. Diseased cells, such as in cancer, co-opt this pathway via CHD1L-driven TCF/LEF transcription to promote survival under therapeutic stress [17, 102, 103]. These findings demonstrate that CHD1L is a key regulator and component of TCF/LEF-driven target gene activation in cancer by promoting chromatin accessibility at target promoters, with context-dependent activity that may differ in non-transformed cells.

Recent studies have shown that CHD1L expression is positively correlated with the presence of myeloid-derived suppressor cells (MDSCs), a cell population known to promote immune evasion by suppressing immune responses [8]. In line with this, the TCF/LEF transcriptional program, where CHD1L plays a key regulatory role, has been shown to inhibit immune surveillance by excluding T cells from the tumor microenvironment and inducing immune checkpoint gene expression [99]. Accordingly, CHD1L functions at the intersection of intrinsic transcriptional programs and extrinsic immunosuppressive cues, especially in disease contexts where immune evasion contributes to pathogenesis.

Collectively, the literature reports and evidence described in this section establish CHD1L as a chromatin remodeler with broad transcriptional influence. In the context of disease, aberrant CHD1L promotes adaptability, survival, and immune evasion under stress and therapeutic pressure, particularly in cancer.

### DNA damage response and repair

**Fig. 5:**
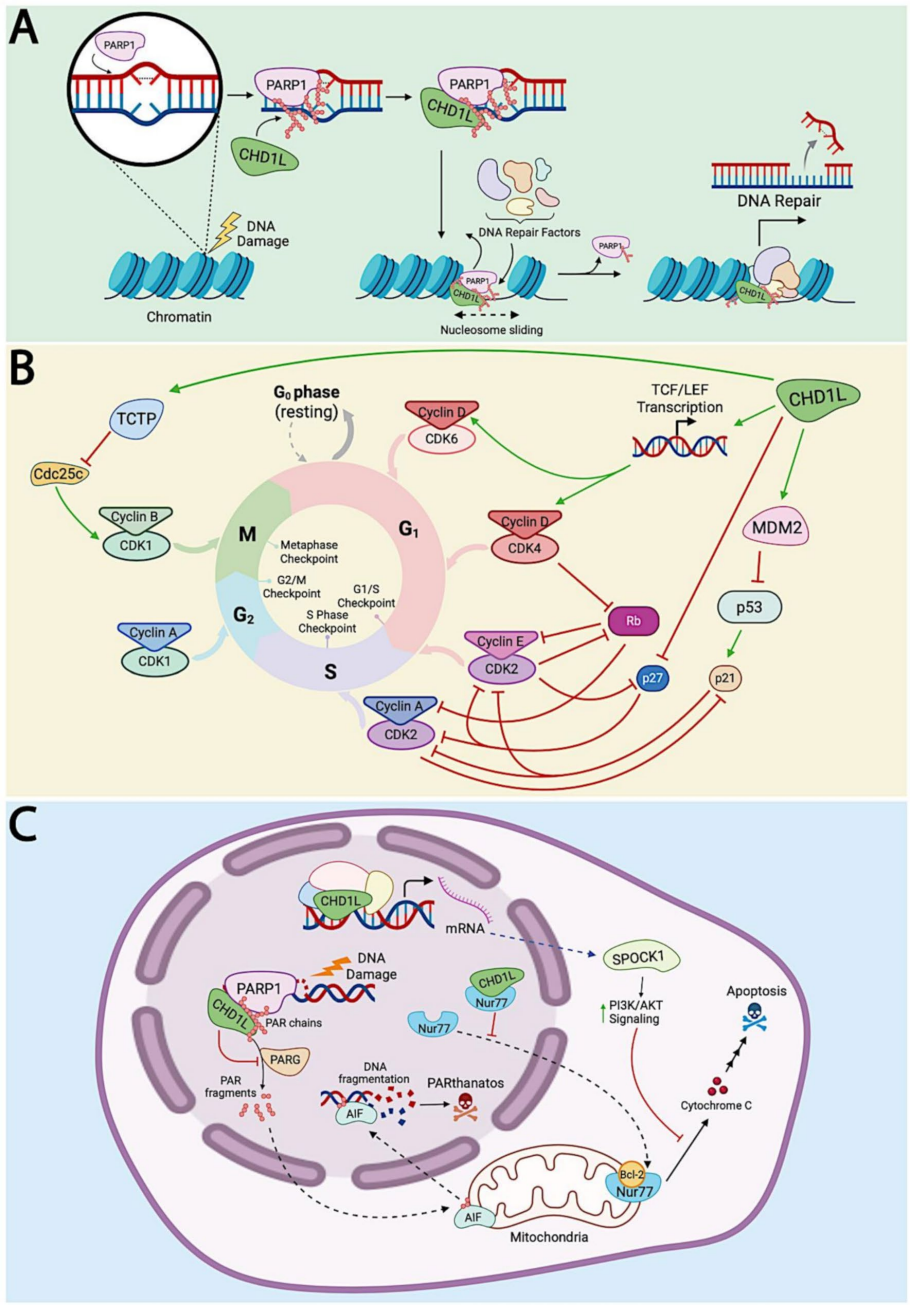
CHD1L regulation of DNA repair and cell cycle progression, and its role as a master regulator of PARthanatos programmed cell death. (**A**) Upon DNA damage, CHD1L is recruited to sites of DNA lesions through its PAR-binding macrodomain following PARP1 activation. CHD1L promotes nucleosome sliding and chromatin relaxation, enabling PARP1 dissociation and the recruitment of downstream DNA repair factors to initiate the DDR. (**B**) CHD1L promotes G_1_/S phase transition by transcriptionally upregulating TCTP, Cyclins D, E, and A, CDK2, CDK4, and CDK6, while repressing p27 and facilitating RB phosphorylation. CHD1L also upregulates MDM2, which destabilizes p53, further promoting cell cycle progression. Green arrows indicate transcriptional activation; red blunt arrows denote repression or inhibition. (**C**) CHD1L suppresses apoptosis by retaining Nur77 in the nucleus and upregulating SPOCK1, which activates the PI3K/Akt pathway to block cytochrome c release from mitochondria. CHD1L also acts as a master regulator of PARthanatos by binding nuclear PAR chains, protecting them from PARG-mediated degradation. This prevents PAR fragmentation and AIF translocation to mitochondria, thereby blocking caspase-independent cell death

One of the most well-understood functions of CHD1L is in the context of DDR and DNA repair [85]. CHD1L is rapidly recruited to DNA lesions through its macro domain, which binds PAR chains synthesized by PARP1 upon sensing DNA damage [104]. PARP1 recognizes both single- and double-strand DNA breaks induced by genotoxic insults such as oxidative stress, UV radiation, ionizing radiation, and DNA damaging agents [105]. Using NAD⁺ as a cofactor, PARP1 synthesizes PAR chains at damage sites, which serve as recruitment signals for DNA repair factors with PAR-binding domains [104]. CHD1L is one such PAR-binding factor, activated through PAR-dependent release of its autoinhibitory macro domain (Fig. 2A) [32, 34, 36, 83, 84, 106]. Once recruited, CHD1L slides nucleosomes near the lesion site, promoting chromatin relaxation and enhancing access for downstream DNA repair complexes (Fig. 5A) [82]. This activity has been shown to be essential for early chromatin remodeling in DDR and is mediated through CHD1L’s ATPase domain, whose function depends on both PAR binding and chromatin engagement [82–84]. Accordingly, knockdown of CHD1L impairs DNA repair efficiency and increases sensitivity to DNA-damaging agents [107].

CHD1L is functionally linked to multiple repair pathways. In base excision repair (BER), which addresses single-base lesions from oxidation, hydrolysis, or alkylation, CHD1L knockdown leads to reduced repair efficiency and increased DNA strand breaks [108]. In nucleotide excision repair (NER), which resolves bulky lesions like those caused by UV irradiation, CHD1L contributes to global genome NER (GG-NER) due to its association with xeroderma pigmentosum linked genes XPC and XPE, which coordinate repair of UV induced DNA damage through the DNA damage recognition complex (DDB2) [106]. Emerging evidence also implicates CHD1L in double-strand break repair. While CHD1L is not a core component of homologous recombination (HR) or non-homologous end joining (NHEJ), it’s chromatin remodeling facilitates access of repair factors to DNA ends. This role is thought to aid the resection and end-joining processes required for both repair pathways, though further investigation is warranted [85].

### Regulation of cell cycle progression

Chromatin restructuring is essential for cellular replication, and therefore post-translational modifications of histones are deeply intertwined with cell cycle control [109]. As such, CHD1L’s role in chromatin relaxation predictably links its activity to mitotic regulation. Indeed, CHD1L has been identified as a promoter of tumor cell proliferation through its mediation of DNA synthesis and G1/S transition [63, 68, 110].

Initiation and progression through cell cycle checkpoints are strictly controlled by regulatory proteins, specifically cyclin-dependent kinases (CDKs). Upregulation of CHD1L leads to an increase in the expression of Cyclins A, D1, and E, as well as CDK2 and CDK4 (Fig. 5B). Conversely, CHD1L downregulates retinoblastoma protein (Rb) and p27 [63, 110]. Activation of CDK4 by D-type cyclins drives the transition from G1 to S phase via phosphorylation of Rb [111]. Rb is a tumor suppressor protein that represses E2F transcription factor targets that would otherwise induce G1/S transition. The inhibitory activity of Rb is neutralized upon phosphorylation by the Cyclin D/CDK4 complex, allowing the expression of E2F targets and the subsequent progression toward S phase [111]. Two of these G1/S transition genes are Cyclins E and A, which bind to and activate CDK2 [112]. CDK2 is normally inhibited by tumor suppressors p21 and p27, but once activated, it promotes degradation of both inhibitors and further phosphorylates Rb [111, 113]. This activity initiates and drives S phase progression by targeting substrates involved in centrosome duplication, histone biosynthesis, and DNA replication [114, 115]. CHD1L’s downregulation of tumor suppressors, along with the upregulation of Cyclins and CDKs that promote G1/S transition, suggests that it drives the cell cycle past this critical checkpoint (Fig. 5B). Likewise, knockdown or small molecule inhibition of CHD1L arrests cells in the G1 phase, further validating its role in this transition [63, 74].

Upregulation of CHD1L has also been associated with modulation of p53 expression and signaling pathways. Several studies have found that CHD1L levels are inversely related to those of p53, a master tumor suppressor [63, 110]. p53 levels are primarily regulated by the mouse double minute two protein (MDM2), which induces p53 ubiquitination and targets it for proteasomal degradation (Fig. 4) [116]. MDM2 appears to be a downstream target of CHD1L, as it is downregulated in CHD1L knockdown lines and upregulated in CHD1L overexpression lines [73]. Since p53 is an upstream transcription factor for p21, a major G1/S regulator, the link between CHD1L and MDM2 further explains why CHD1L promotes G1/S phase transition (Fig. 5B) [117]. However, p53 also regulates the G2/M checkpoint, so CHD1L-mediated p53 suppression may facilitate cell cycle progression through multiple phases.

CHD1L may also play a role in mediating mitotic exit. It has been reported that CHD1L induces expression of TCTP, which leads to degradation of Cdc25C [97]. This results in increased Cdk1 phosphorylation and a corresponding decrease in its activity, ultimately promoting mitotic exit (Figs. 4 and 5B). In hepatocellular carcinoma, CHD1L upregulation has been shown to accelerate mitotic exit to the extent that it causes chromosome mis-segregation and increases overall mutational burden [97]. Taken together, these findings suggest that CHD1L is a multifaceted regulator of the cell cycle, acting at both early and late phases to promote division and proliferation.

### Role in programmed cell death

Among its diverse roles in development and disease, a major function of CHD1L is that of a master regulator of cell survival, promoting cellular repair and suppressing multiple forms of programmed cell death. It modulates checkpoint proteins involved in cell cycle progression and immune signaling, governs DDR and repair pathways, and orchestrates transcriptional programs and protein-protein interactions that favor survival over cell death. This section highlights how CHD1L suppresses both apoptotic and non-apoptotic death mechanisms through interactions with key regulators (Fig. 5C).

Arguably the most well-characterized form of cell death suppressed by CHD1L is apoptosis. CHD1L is a multifaceted anti-apoptotic factor that inhibits pro-apoptotic proteins Nur77 and TP53, while activating the anti-apoptotic factors SPOCK1, TCTP, and MDM2 (Figs. 4 and 5C) [28, 118]. Among these, the interaction between CHD1L and the orphan nuclear receptor Nur77 (NR4A1) is particularly well-defined. Nur77 translocates from the nucleus to the mitochondria in response to apoptotic stimuli, where it binds Bcl-2 and induces a conformational change that triggers cytochrome c release, Apaf-1 activation, and caspase-mediated apoptosis (Fig. 5C) [119, 120]. CHD1L’s C-terminal macrodomain binds Nur77 in the nucleus, preventing this critical translocation step and thereby suppressing the mitochondrial apoptotic cascade [118]. Functional experiments in hepatocellular carcinoma cells demonstrate that CHD1L overexpression blocks Nur77 translocation, inhibits cytochrome c release, and prevents caspase-9 and caspase-3 activation. Conversely, silencing CHD1L restores Nur77-mediated apoptosis, confirming that CHD1L serves as a key cellular safeguard against apoptotic signaling.

CHD1L also suppresses apoptosis through modulation of the MDM2–p53 axis. As the primary negative regulator of p53, MDM2 binds to p53, blocks its transcriptional activity, and targets it for proteasomal degradation (Fig. 4) [121]. In breast cancer models, CHD1L transcriptionally upregulates MDM2, as shown by cDNA microarray analysis and confirmed via Western blot, where CHD1L overexpression increased MDM2 protein levels, and CHD1L knockdown reduced them [73]. In vivo, CHD1L overexpression in mouse tumors led to increased MDM2 staining by immunohistochemistry, while CHD1L silencing resulted in marked downregulation of MDM2 expression. This upregulation of MDM2 destabilizes p53 and inhibits its ability to activate downstream pro-apoptotic targets. Thus, by promoting MDM2 expression and suppressing TP53, CHD1L enhances tumor cell survival and proliferation while disabling key apoptotic checkpoints, further supporting its role as a central suppressor of programmed cell death in cancer.

CHD1L promotes tumor cell survival by upregulating SPOCK1 (see Control of Gene Expression and Signaling above), a matricellular glycoprotein that blocks apoptosis through activation of the PI3K/Akt signaling pathway (Fig. 4 C). CHD1L-driven SPOCK1 expression enhances Akt phosphorylation, which inhibits mitochondrial cytochrome c release and suppresses downstream activation of caspase-9 and caspase-3. In hepatocellular carcinoma cells, knockdown of CHD1L induces apoptosis, as shown by increased Annexin V staining, mitochondrial membrane depolarization, and elevated levels of cleaved PARP and caspase-3. This apoptotic phenotype is accompanied by reduced SPOCK1 expression and is rescued by ectopic SPOCK1 overexpression, confirming that SPOCK1 mediates CHD1L-dependent survival. CHD1L-induced SPOCK1 expression also contributes to tumor progression, with elevated SPOCK1 levels correlating with increased metastasis, advanced tumor stage, and poor clinical outcomes for cancer patients [78].

One of the most striking recent discoveries is that CHD1L suppresses PARthanatos (Fig. 5C) [76], a caspase-independent form of programmed cell death first reported in 2009 [122]. PARthanatos can be initiated by oxidative stress, hyperactivated PARP1, or nuclear PAR signaling that leads to PAR polymer hydrolysis and fragmentation. These PAR chains are normally degraded PAR-glycohydrolase (PARG), releasing free PAR fragments into the cytoplasm, where they bind apoptosis-inducing factor (AIF) localized in the mitochondria. PAR fragment binding triggers AIF release and translocation to the nucleus, where it induces massive DNA fragmentation and initiates PARthanatos [123, 124]. AIF translocation to the nucleus is considered a well-established biomarker of PARthanatos and is routinely used to confirm pathway activation in both in vitro and in vivo models [125].

CHD1L directly binds PAR chains in the nucleus via its macrodomain and shields them from PARG-mediated hydrolysis, effectively blocking the upstream trigger of AIF release and suppressing PARthanatos (Fig. 5C) [76]. This protective interaction prevents cytoplasmic PAR accumulation, mitochondrial depolarization, and AIF translocation to the nucleus. In breast and colorectal cancer models, pharmacological inhibition of CHD1L results in the entrapment of CHD1L on chromatin, deprotection of nuclear PAR chains, and robust induction of PARthanatos, as measured by AIF translocation to the nucleus [76].

Together, these findings establish CHD1L as a central suppressor of both apoptotic and non-apoptotic forms of programmed cell death. By disabling key cell death checkpoints—including Nur77 mitochondrial signaling, the MDM2–TP53 axis, and PAR-mediated AIF activation—CHD1L enables cancer cells to survive under conditions of metabolic stress, immune surveillance, and genotoxic therapy. The multifaceted survival advantages conferred by CHD1L likely contribute to the aggressive behavior and treatment resistance observed in many cancers where CHD1L is implicated. As such, CHD1L has emerged as a master regulator of PARthanatos and a gatekeeper of cancer cell viability under stress.

## Therapeutic strategies targeting CHD1L

The role of CHD1L in tumor progression, cell survival, metastasis, and MDR makes it an attractive molecular target for the next generation of cancer treatments. Knockdown of CHD1L dramatically reduces the aggression and growth of cancerous cells, while concurrently sensitizing them to chemotherapy. This indicates the strategy of CHD1L suppression could be valid as either a single agent therapy or combination therapy. Although there are no FDA approved CHD1L-related treatments on the market to date, there are several that have been proposed or are currently in development that may well drive forward the field of targeted cancer therapy [18].

### Gene therapy and genome editing approaches

One proposed strategy to fight the malignant activity conferred by CHD1L in cancer is to prevent its expression using gene therapy. Gene editing approaches such as CRISPR/Cas9 have been explored to eliminate CHD1L expression, both in vitro and in vivo, revealing downstream signaling effects but have not yet been implemented to treat patients in the clinic. Tumor cells with CHD1L knockout display dramatically less aggressive metastatic behavior and are sensitized to PARP inhibitors (PARPi) [14, 15, 126, 127]. Although CRISPR/Cas9 therapy has shown potential for personalized cancer treatment in preliminary studies through the knockout of oncogenes, challenges remain, including off-target activity and unintended genomic alterations [128].

Another method of gene silencing is RNA interference, or RNAi, which utilizes one of two non-coding RNA strands to knock down target genes, short hairpin RNA (shRNA) or small interfering RNA (siRNA). shRNA silencing is generally preferred over the siRNA approach for long term gene silencing because shRNA is more stable and is capable of DNA integration, so it can generate stable knockdown cell lines [129]. The shRNA method involves transducing small hairpin RNA (shRNA) into cancer cells using a lentiviral vector [77]. The shRNA molecule corresponds with the mRNA strand for CHD1L, and will target it for degradation by the endogenous RNA induced silencing complex (RISC) [129]. The knockdown of CHD1L using shRNA suppressed proliferation, motility, invasion, and survival in several different cancer lines [17, 77]. Although both RNAi strategies have been utilized to study the cellular function of CHD1L in vitro, it has yet to be effectively validated as a form of cancer treatment in humans. However, the use of CHD1L genetic silencing in future cancer treatment has been proposed as a potential therapeutic avenue [77].

Should RNAi be utilized in cancer treatment, an effective delivery method must be employed, as free RNA particles are quickly degraded and may cause toxicity in circulation [130]. The most popular RNAi delivery systems in use at the moment are lipid nanoparticles and viral vectors [131]. The use of shRNA and siRNA gene therapy against oncogenes has proven effective in animal models, although the feasibility and safety of this method is still unclear [132]. Nonetheless, if RNAi gene silencing becomes a clinically valid therapeutic strategy in human disease, CHD1L will be a strong target candidate for cancer treatment.

### Small molecule inhibitors of CHD1L (CHD1Li)

An alternative to gene therapy for targeting CHD1L in cancer is the use of small molecule inhibitors. The first CHD1L inhibitors (CHD1Li) were identified in 2020 through high-throughput screening (HTS) of chemical libraries against a truncated, catalytically active form of CHD1L (cat-CHD1L) [18, 20]. These inhibitors demonstrated the ability to suppress CHD1L-mediated TCF/LEF transcriptional activity, reverse EMT, and reduce cancer stem cell (CSC) stemness in colorectal cancer models, mirroring the effects observed with CHD1L knockdown. Unlike CHD1L knockdown, small molecule CHD1Li potently induced programmed cell death, which was initially thought to be via the induction of apoptosis but later shown to be PARthanatos [18, 75, 76]. Furthermore, Abbott et al., characterized CHD1L as a required component of TCF/LEF-transcription in colorectal cancer cells (Fig. 4) [18]. Notably, TCF/LEF-transcription is known to be a master regulator of EMT and cancer stem cell stemness [133], which is consistent with CHD1Li induced reversion of EMT and suppression of CSC stemness. Medicinal chemistry optimization of these initial leads led to the development of CHD1Li with improved potency, drug-like properties, and oral bioavailability [94]. These results continue to support CHD1L as a viable target for small molecule drug development.

From this medicinal chemistry campaign, OTI-611 emerged as the lead analog, demonstrating low micromolar to nanomolar cytotoxicity across multiple cancer cell lines and a favorable PK profile in mice [94]. While originally developed for CRC, OTI-611 has also shown efficacy in breast cancer [76]. The comprehensive mechanism of action (MOA) for OTI-611 and its analogs has been elucidated and involves allosteric binding to CHD1L, inhibiting its ATPase activity and promoting the entrapment of CHD1L on chromatin [75]. Once entrapped, CHD1L can no longer mediate chromatin remodeling, leading to the suppression of malignant gene expression, DDR, and cell cycle progression [74–76].

Entrapment of CHD1L also interferes with its ability to bind PAR chains in the nucleus, leaving them vulnerable to hydrolysis by PARG. The resulting PAR fragments translocate to the cytoplasm, where they bind to AIF, triggering its release and subsequent translocation to the nucleus, ultimately inducing PARthanatos (Fig. 5C) [75, 76]. Importantly, this MOA differs significantly from the effects of CHD1L knockdown or knockout. While genetic disruption produces viable tumor cells with impaired function [10, 11, 91], pharmacological inhibition with CHD1Li OTI-611 and its analogs induces potent tumor cell death, underscoring a critical distinction between therapies that promote protein loss and traditional small molecule inhibition. Albeit no CHD1L-targeting PROTACs (Proteolysis-targeting chimeras) have been developed to date, such strategies would likely phenocopy gene depletion rather than mimic the lethal mechanism achieved by small molecule CHD1Li [134].

In addition to the CHD1Li identified through HTS and optimized in this program, several other groups have reported CHD1L-targeting compounds. Eisbach Bio has developed EIS-12,656, a CHD1Li that binds to an allosteric pocket within the N-ATPase domain [135], differing from the binding mode of OTI-611 and its analogs [20, 94]. Eisbach Bio’s CHD1Li, EIS-12,656, is currently being evaluated in phase 1/2 clinical trials (ClinicalTrials.gov: NCT06525298). This open label trial will investigate the use of EIS-12,656 in advanced stages of cancer with homologous recombination deficiencies such as BRCA1/2 mutations. Phase 1 involves administering increasing doses of EIS-12,656 to assess the safety and establish a tolerable dose for phase 2. In phase 2 patients will receive a daily dose of the CHD1Li either alone or in combination with PARPi or Trastuzumab deruxtecan, both FDA approved cancer drugs. This trial aims to evaluate the tolerability and effectiveness of EIS-12,656 alone or in combination with SOC therapies.

Separately, a study utilizing artificial intelligence–driven virtual screening and molecular modeling identified a series of CHD1Li, from which three compounds, C071–0684, K284–5877, and K284–5881, were prioritized [19]. Of these molecules, CO71-0684 was the most potent, demonstrating inhibitory activity against the cat-CHD1L enzyme. Modeling studies suggest that these CHD1Li engage the N-ATPase lobe active site, classifying them as competitive inhibitors. Notably, these inhibitors also induced cytotoxicity in breast and colorectal cancer cell lines.

### CHD1Li combination therapies with chemotherapy and targeted agents

Pharmacological inhibition of CHD1L has demonstrated remarkable synergy with SOC chemotherapies. Notably, CHD1Li OTI-611 significantly enhances the cytotoxic potency of prodrug irinotecan (SN-38, active metabolite), 5-fluorouracil (5-FU), doxorubicin, oxaliplatin, and etoposide in both colorectal and breast cancer models [74–76]. In colorectal cancer organoids, OTI-611 improved the potency of SN-38 by nearly 1000-fold and 5-FU by 500-fold in chemoresistant SW948 models [75]. This synergy translated in vivo, where combination therapy with OTI-611 and irinotecan led to near-complete tumor regression and nearly tripled survival compared to irinotecan alone. Mechanistically, this synergy was linked to OTI-611’s ability to entrap CHD1L on chromatin, which reprograms the cellular response to chemotherapy. Instead of inducing their typical mechanisms of action such as apoptosis or cell cycle arrest, chemotherapy combined with OTI-611 triggered G1 arrest and PARthanatos. This shift was consistent across all chemotherapies tested [74–76], and may explain OTI-611’s ability to overcome MDR pathways commonly associated with chemotherapy failure.

CHD1Li also exhibit strong synergy with PARPi, particularly olaparib. In BRCA-mutant cancers, PARPi induce synthetic lethality by trapping PARP1/2 at DNA damage sites, disrupting the DDR, and leading to replication stress and apoptosis [127, 136]. However, CHD1L has been shown to promote PARPi resistance in BRCA-mutant cells, and CRISPR knockout of CHD1L restores PARPi sensitivity in vitro and in vivo [15]. Pharmacological CHD1L inhibition with OTI-611 similarly enhances PARPi efficacy, promoting PARP1/2 trapping and PARthanatos in both BRCA-mutant and BRCA-wildtype breast cancer models [74, 76]. Eisbach Bio’s CHD1Li EIS-12,656 has also shown preclinical synergy with PARPi [130]; however, it has not yet been reported to reprogram the mechanism of PARPi-induced cell death as observed with OTI-611. CHD1Li may also improve responses to other targeted therapies. He et al. recently demonstrated that OTI-611 synergized with the tyrosine kinase inhibitor sunitinib in renal cancer models in vitro and in vivo [2]. These findings broaden the therapeutic scope of CHD1L inhibition to include kinase inhibitors, further underscoring the potential utility in combination therapy.

In summary, several groups are advancing CHD1L-targeting strategies into preclinical and clinical development. CHD1L remains a compelling oncology target due to its central role in tumor progression, metastasis, cell survival, and MDR. While multiple CHD1Li have demonstrated promising preclinical activity, mechanistic studies of OTI-611 have provided the most detailed evidence to date of CHD1L-dependent reprogramming of tumor cell death. Among currently reported CHD1Li, OTI-611 and its analogs are the only agents shown to synergize with chemotherapy, PARPi, and other targeted therapies that do not cause DNA damage, while shifting tumor cell death pathways toward PARthanatos through chromatin entrapment. These findings highlight the potential of CHD1L inhibition not only as a standalone therapeutic strategy but also as a foundation for rational drug combinations that overcome resistance mechanisms in cancer.

## Conclusion

CHD1L is a multifunctional chromatin remodeler with essential roles in embryonic development, DNA repair, transcriptional regulation, and cellular stress responses [18, 39, 74]. Its activity is tightly regulated under normal physiological conditions, but disruption of this regulation has been implicated in a growing number of human diseases. In addition to early embryonic lethality observed in knockout models, rare missense mutations in CHD1L have been linked to congenital anomalies affecting renal and reproductive development [44, 45]. Moreover, its involvement in chromatin remodeling and immune signaling suggests possible roles in inflammatory and stress-related disorders, though these areas remain underexplored.

CHD1L’s function in cancer is the most extensively studied. Overexpression of CHD1L is common across a wide range of solid tumors and is strongly associated with tumor progression, metastatic potential, and poor prognosis [4, 8, 67]. Mechanistically, CHD1L promotes tumor growth through modulation of DNA damage repair, activation of survival and cell cycle pathways, regulation of EMT, and suppression of both apoptotic and non-apoptotic forms of cell death [18, 28, 76]. Importantly, its low expression in most normal tissues and cancer-specific upregulation position CHD1L as an attractive target for cancer drug development [75]. As CHD1Li advance in development, a new class of therapies may emerge that can overcome MDR and restore treatment sensitivity in aggressive, refractory cancers. Continued exploration of CHD1L’s roles across both normal and disease states will not only advance the basic understanding of chromatin dynamics but also drive the next generation of targeted cancer therapies.

## Data Availability

No datasets were generated or analysed during the current study.

## Abbreviations

ABCB1: ATP-Binding Cassette Sub-Family B Member
ADHD: Attention Deficit/Hyperactivity Disorder
ADP: Adenosine Diphosphate
AIF: Apoptosis Inducing Factor
Akt: Protein Kinase B
ALC1: Amplified in Liver Cancer 1
AMP: Adenosine Monophosphate
ARHGEF9: Rho Guanine Nucleotide Exchange Factor 9
ARK5: AMPK-Related Protein Kinase 5
ATP: Adenosine Triphosphate
ATPase: Adenosine Triphosphatase
Bcl-2: B-Cell Lymphoma 2
BER: Base Excision Repair
BRCA: Breast Cancer Gene
BRD4: Bromodomain-Containing Protein 4
c-MYC: Cellular Myelocytomatosis Oncogene
CAKUT: Congenital Anomalies of the Kidney and Urinary Tract
Cdc25C: Cell Division Cycle 25 C
Cdc42: Cell Division Cycle protein 42
CDK: Cyclin Dependent Kinase
CHD1: Chromodomain Helicase DNA Binding Protein 1
CHD1L: Chromodomain Helicase DNA Binding Protein 1-Like
CHD1Li: CHD1L Inhibitor(s)
CHD: Chromodomain helicase DNA-binding
CRISPR/Cas9: Clustered Regularly Interspaced Short Palindromic Repeats / CRISPR-Associated Protein 9
CSC: Cancer Stem Cell
DDB2: DNA Damage Binding Protein-2
DDR: DNA Damage Response
DNA: Deoxyribonucleic Acid
E2F: Early Region 2 Binding Factor
EMT: Epithelial-mesenchymal Transition
EPO: Erythropoietin
FOXO3a: Forkhead Box O3
GDNF: Glial Cell Line Derived Neurotrophic Factor
GG-NER: Global Genome Nucleotide Excision Repair
H2A/H2B: Histone 2 A Subunit/Histone 2B Subunit Dimer
H4: Histone H4 Subunit
HCC: Hepatocellular Carcinoma
HELICc: Helicase Superfamily C-Terminal Domain
HIF: Hypoxia-Inducible Factor
HIV-1: Human Immunodeficiency Virus-1
HR: Homologous Recombination
HTS: High Throughput Screening
kDa: Kilodalton
KLF4: Krüppel-Like Factor 4
LOX: Lysyl Oxidase
MDA: Müllerian Duct Abnormalities
MDM2: Mouse Double Minute 2 Protein
MDR: Multi-Drug Resistance
miRNA: MicroRNA
MMP2: Matrix Metalloproteinase 2
mRNA: Messenger Ribonucleic Acid
MS: Multiple Sclerosis
mTOR: Mammalian Target of Rapamycin
N-ATPase/C-ATPase: N-terminal/C-Terminal ATPase
NAD+: Nicotinamide Adenine Dinucleotide
NANOG: Nanog Homeobox protein
NDNF: Neuron Derived Neurotropic Factor
NER: Nucleotide Excision Repair
Nur77 (NR4A1): Nuclear Receptor Subfamily 4 Group A Member 1
OCT4: Octamer-binding Transcription Factor 4
PAR: Poly(ADP-ribose)
PARG: Poly(ADP-Ribose) Glycohydrolase
PARP1/2: Poly(ADP-ribose) Polymerase 1/2
PARPi: PARP Inhibitor(s)
PAX6: Paired Box 6
PI3K: Phosphoinositide 3-Kinase
PPMS: Progressive Multiple Sclerosis
PRKAB2: Protein Kinase AMP-Activated Non-Catalytic Subunit Beta 2
Rb: Retinoblastoma protein
RCC: Renal Cell Carcinoma
RISC: RNA Induced Silencing Complex
RLS: Regulatory Linker Segment
RNAi: RNA Interference
shRNA: Short Hairpin RNA
siRNA: Small Interfering RNA
SIRT7: Sirtuin 7 Deacetylase
SNF-2: Sucrose Non-Fermentation 2
SOX2: Sex Determining Region Y-Box 2
SPOCK1: Sparc/Osteonectin, Cwcz and Kazal-like Domain Proteoglycan 1
SSC: Spermatogonial Stem Cells
TCF/LEF: T-cell Factor/Lymphoid Enhancer Factor
TCTP: Translationally Controlled Tumor Protein
VEGFA: Vascular Endothelial Growth Factor A
VSMC: Vascular Smooth Muscle Cells
ZKSCAN3: Zinc Finger with KRAB and SCAN domain 3
